# COVID-19: Rational discovery of the therapeutic potential of Melatonin as a SARS-CoV-2 main Protease Inhibitor

**DOI:** 10.7150/ijms.48053

**Published:** 2020-07-30

**Authors:** Eduardo L Feitosa, Francisco Tiago Dos S S Júnior, José Arimatéa De O Nery Neto, Luis F L Matos, Matheus H De S Moura, Thiele Osvaldt Rosales, Guilherme Barroso L De Freitas

**Affiliations:** 1 Laboratório de Química Medicinal e Biotecnologia (LAQUIMB), Department of Biochemistry and Pharmacology, Federal University of Piauí, Teresina, PI, Brazil.; 2 Department of Pharmacology, Federal University of Santa Catarina, SC, Brazil.

**Keywords:** Melatonin, COVID-19, Rational Design, Docking, hyper-inflammation

## Abstract

The SARS-CoV-2 spread quickly across the globe. The World Health Organization (WHO) on March 11 declared COVID-19 a pandemic. The mortality rate, hospital disorders and incalculable economic and social damages, besides the unproven efficacy of the treatments evaluated against COVID-19, raised the need for immediate control of this disease. Therefore, the current study employed *in silico* tools to rationally identify new possible SARS-CoV-2 main protease (Mpro) inhibitors. That is an enzyme conserved among the coronavirus species; hence, the identification of an Mpro inhibitor is to make it a broad-spectrum drug. Molecular docking studies described the binding sites and the interaction energies of 74 Mpro-ligand complexes deposited in the Protein Data Bank (PDB). A structural similarity screening was carried out in order to identify possible Mpro ligands that show additional pharmacological properties against COVID-19. We identified 59 hit compounds and among them, melatonin stood out due to its prominent immunomodulatory and anti-inflammatory activities; it can reduce oxidative stress, defence cell mobility and efficiently combat the cytokine storm and sepsis. In addition, melatonin is an inhibitor of calmodulin, an essential intracellular component to maintain angiotensin-converting enzyme 2 (ACE-2) on the cell surface. Interestingly, one of the most promising hits in our docking study was melatonin. It revealed better interaction energy with Mpro compared to ligands in complexes from PDB. Consequently, melatonin can have response potential in early stages for its possible effects on ACE-2 and Mpro, although it is also promising in more severe stages of the disease for its action against hyper-inflammation. These results definitely do not confirm antiviral activity, but can rather be used as a basis for further preclinical and clinical trials.

## Introduction

The recent outbreak of COVID-19 has become a pandemic with millions of infected patients and tens of thousands of deaths worldwide 1. COVID-19 is an infectious disease caused by the novel coronavirus classified as 2019-nCoV and named Severe Acute Respiratory Syndrome Coronavirus-2 (SARS-CoV-2 or merely CoV-2), a highly contagious RNA virus 2. The severity of COVID-19 symptoms can range from very mild, cold-like symptoms to most people, to a severe disease that requires hospitalization and oxygen support to approximately 10% of those infected, with 5% requiring admission to an intensive care unit 3,4.

Currently, there is no specific drug treatment for COVID-19. Some therapeutic options are antiviral drugs (lopinavir 5-7, danoprevir 8, ritonavir 5, 9, remdesivir 10, 11, umifenovir 12, 13), immunosuppressants (iltuximab 14, baricitinib 15, meplazumab 16, bevacizumab 17, 18, tocilizumab 19-22, glucocorticoids 23-26), chloroquine and hydroxychloroquine 27-31, antipyretics and mechanical respiratory support 32. Clinical trials investigate the efficacy of these drugs and seek to define the most appropriate moment, therapeutic doses and patients to initiate the drug intervention 33.

Up to the present date, no drugs or vaccines have confirmed their clinical efficacy against COVID-19. The results indicated therapeutic ineffectiveness in most clinical studies or low resolution of prognosis, such as mortality and length of hospital stay 5, 34. The lack of double-blind, randomized, placebo-controlled trials with a correct sample size necessary to get accurate and inference worthy results are other observed problems 35, 36. Research groups, biotech start-ups and large industries have come together or individually as part of an international collaboration to help hasten the availability of a vaccine against COVID-19 37. However, a vaccine for general use takes time to develop and it is not likely to be available to the entire population before January 2021.

One factor that negatively influences success in drug discovery and development is the absence of a rational design of biologically active molecules against possible therapeutic targets identified in the viral life cycle of CoV-2 38. Among the various therapeutic targets, the SARS-CoV-2 main protease (Mpro, also called chymotrypsin-like protease, 3CLpro) was structured and repositioned in the Protein Data Bank (PDB) 39, 40. It stands out as a potential target for the inhibition of CoV replication, but it still lacks rational studies to search for possible inhibitors.

There is an urgent need for effective treatment and prevention strategies to contain the progress of cases and, consequently, of mortality. Therefore, we present a rational search for Mpro inhibitors, which used molecular modelling tools and results from previous clinical and preclinical studies.

## Materials and Methods

**Figure 1:**
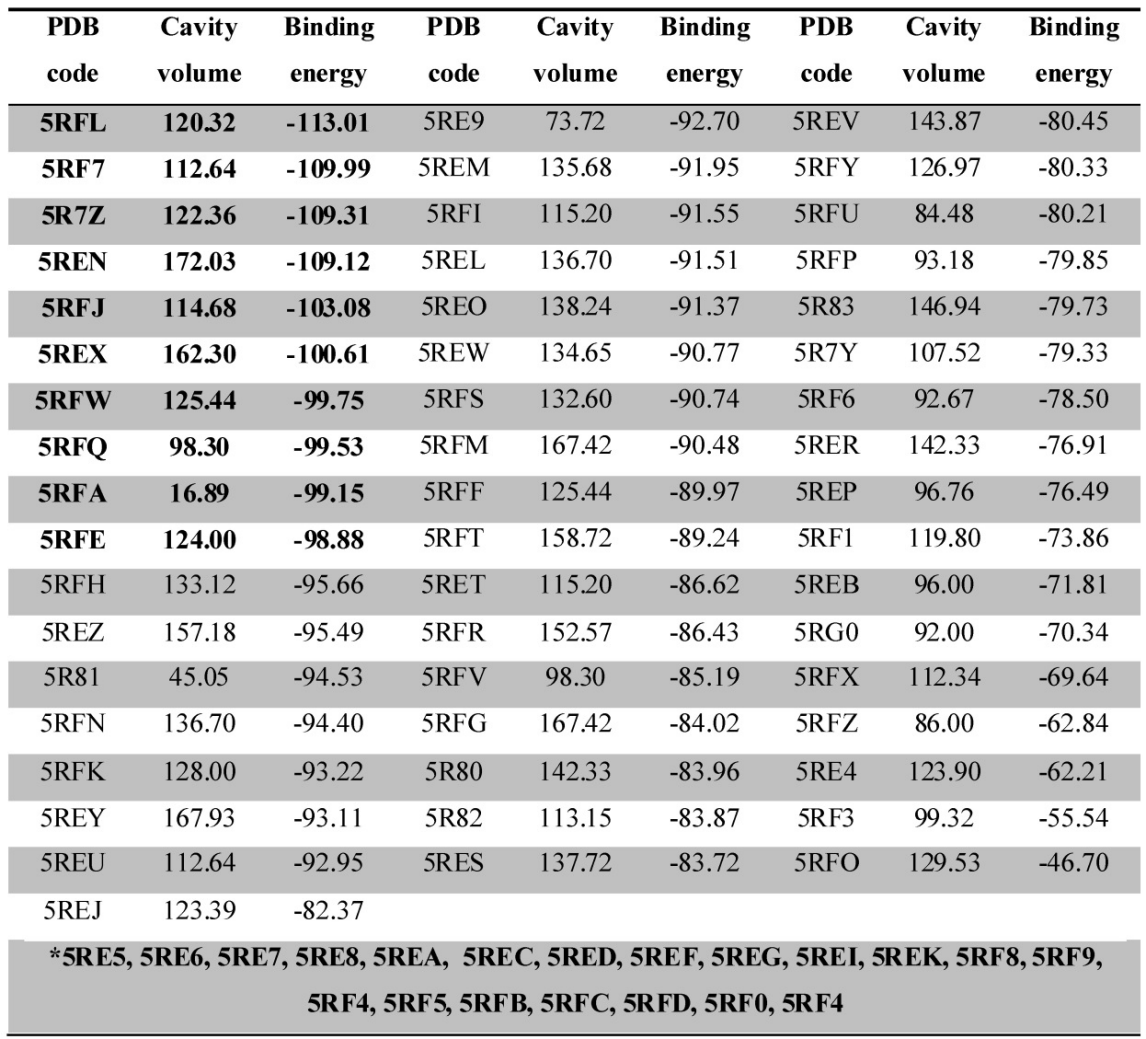
Schematic representation of the work sequence followed in this study.

**Figure 1** summarizes the workflow of the methodology used in this study as detailed later.

### Analyses and validation of CoV-2 main protease complexed with ligand

The protein-ligand complexes for Mpro were taken from PDB (http://www.rcsb.org). We evaluated the amount of amino acid residues, presence and localization of gaps and resolution (Å) of deposited complexes. MolProbity Server calculated bond lengths and bond angles of standard protein residues, too-close contacts, Ramachandran outliers and rotamer outliers of 74 complexes of Mpro present in the Protein Data Bank 41.

### Molecular docking

Initially, a subset of 74 poised fragments was retrieved from its corresponding PDB entries of ligand-Mpro complexes for structure-based docking.

WaveFunction Spartan 14 (v1. 1.4) was employed to analyze and correct possible errors in the structure of binders, partial charges and electrostatic surfaces. The Molegro Virtual Docker (MVD, version 5.5) and Discovery Studio (DS, version 3.5, Accelrys Software Inc., San Diego) pieces of software were utilized to calculate the partial charges of protein-ligand complexes, add hydrogen atoms, optimize hydrogen bonds and remove atomic clashes 42.

The size and position of the Mpro potential binding sites (also referred to as cavities or active sites) and physicochemical properties of protein surface were identified using the built-in cavity detection algorithm with atom probe size of 1.2Å. The positioning of binders in the crystal served as a reference as possible binding sites. Internal electrostatic interactions (ES), hydrogen bond, intramolecular torsion energies and MolDock Optimizer algorithm were used to measure the interaction energies (Kcal / mol). The default settings for the docking search algorithm were applicable for the other parameters.

The docking complexes were ranked based on the energy of interaction, Table 1. The ligand-Mpro interactions, changes in the volume of the binding site and amino acids present in the interaction site were evaluated three-dimensionally with the MVD software and in 2D with the DS software. Specific amino acids and chain side of ligand involved in ligand/protein interactions were also confirmed and explored on a structure activity relationship (SAR).

After identifying the residues of the interaction site within a 9Å radius, we searched for likely proteins with high density and similarity in the active site, aiming at understanding how conserved the residues around the ligand are. For so, the alignment of the FASTA sequence of all the Mpro was assessed by the Blast and T-COFFEE servers and the identity score generated by T-COFFEE was used as the measuring parameter.

### Similarity search

The search for structural similarity used the 10 hit molecules that presented the best interaction energies (Kcal/mol) measured in the docking study among all 74 ligand-Mpro complexes from PDB. For the similarity search for ligands of Mpro, we used the Swisssimilarity database 43, 44. The cutoff criteria for the selection were molecules with available *in vivo* studies, clinical studies and similarity index greater than 0.7, on a scale determined by the Swiss Similarity server up to 1.0. In order to determine the drug-likeness of the hits identified, molecular hydrophobicity parameter LogP, molecular weight (MW), the total polar surface area (TPSA) and Lipinski's rule of five (Ro5) were calculated using Swiss ADME 44.

### Molecular docking of hits and selected analogues

The selected hits (top 10 best-scored compounds identified by previous docking study), as well as their respective similar binders, were docked into SARS-CoV-2 main protease (Mpro) with unliganded active site (PDB id: 6Y84). Once again, the docking simulations were performed with MVD using the same protocol previously optimized in terms of scoring function, flexible ligand, binding site and radius of the binding site (6 Å), with the presence of crystallographic water within the binding site.

## Results and Discussion

The majority (92%) of the 72 complexes studied were characterized as high-resolution structures (< 2 Å). We have more confidence in the location of atoms in structures with these resolution values. In all complexes, the gaps were located only in the protease loop structures, away from the binding site and the total residues were between 304 and 306 conserved amino acids solved by the X-ray diffraction (XRD) method. MolProbit results confirmed acceptable characteristics of complexes, i.e., in Ramachandran plot, more than 97.6% residues were in a favoured region, which is close to the requisite 98% for validating a 3D structure. The parameters of rotamers, Cβ deviations, Cis Prolines and CaBLAM outliers also reinforced the tridimensional quality of proteins 45.

From the docking results, we were able to identify the binding energy (Kcal/mol) between Mpro and ligands (**Table 1**) and active-site residues of amino acids. These results recognized top 10 complexes (highlighted in bold) with greatest binding energy and their respective ligands were characterized as potential hits.

**Figure 2:**
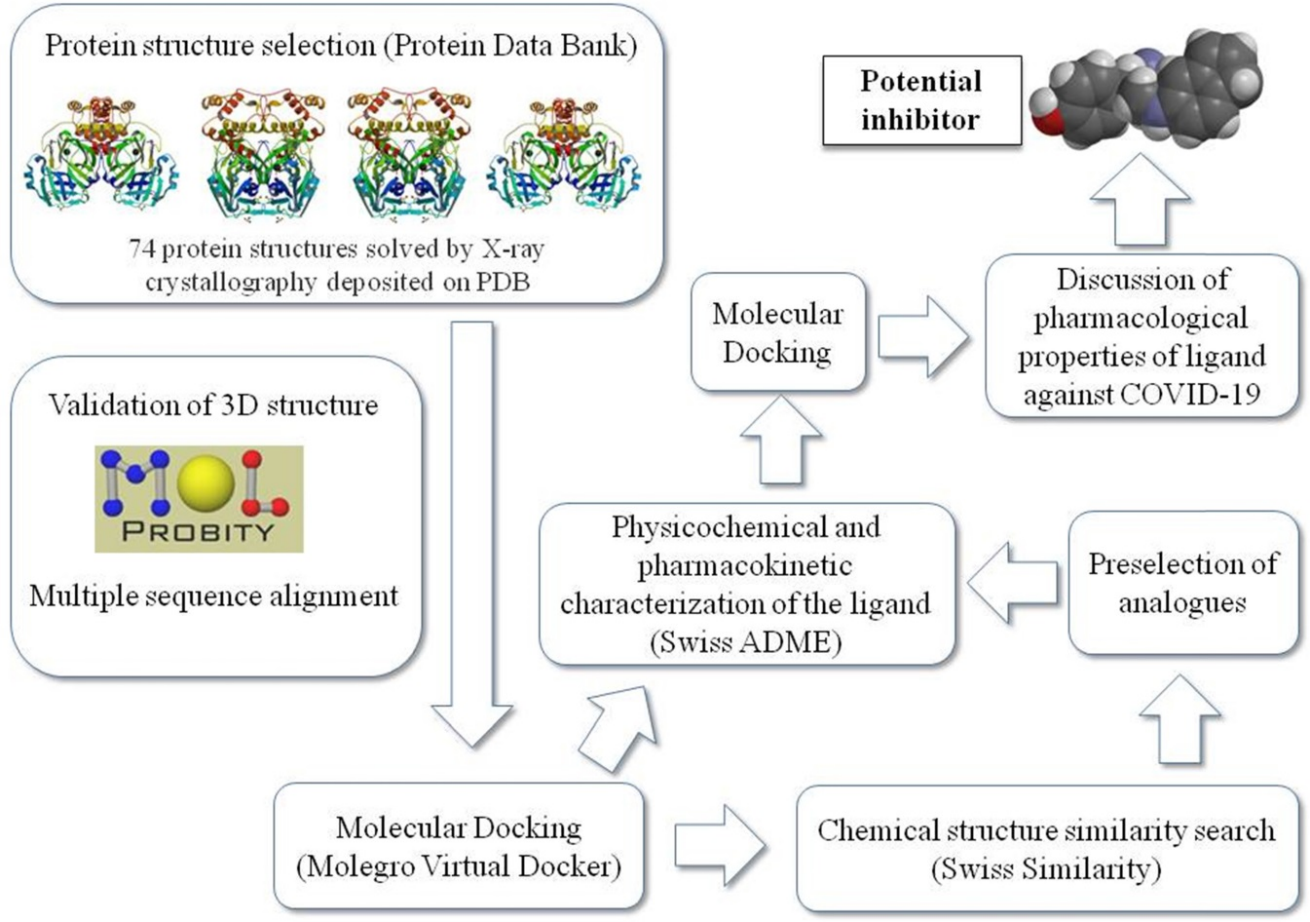

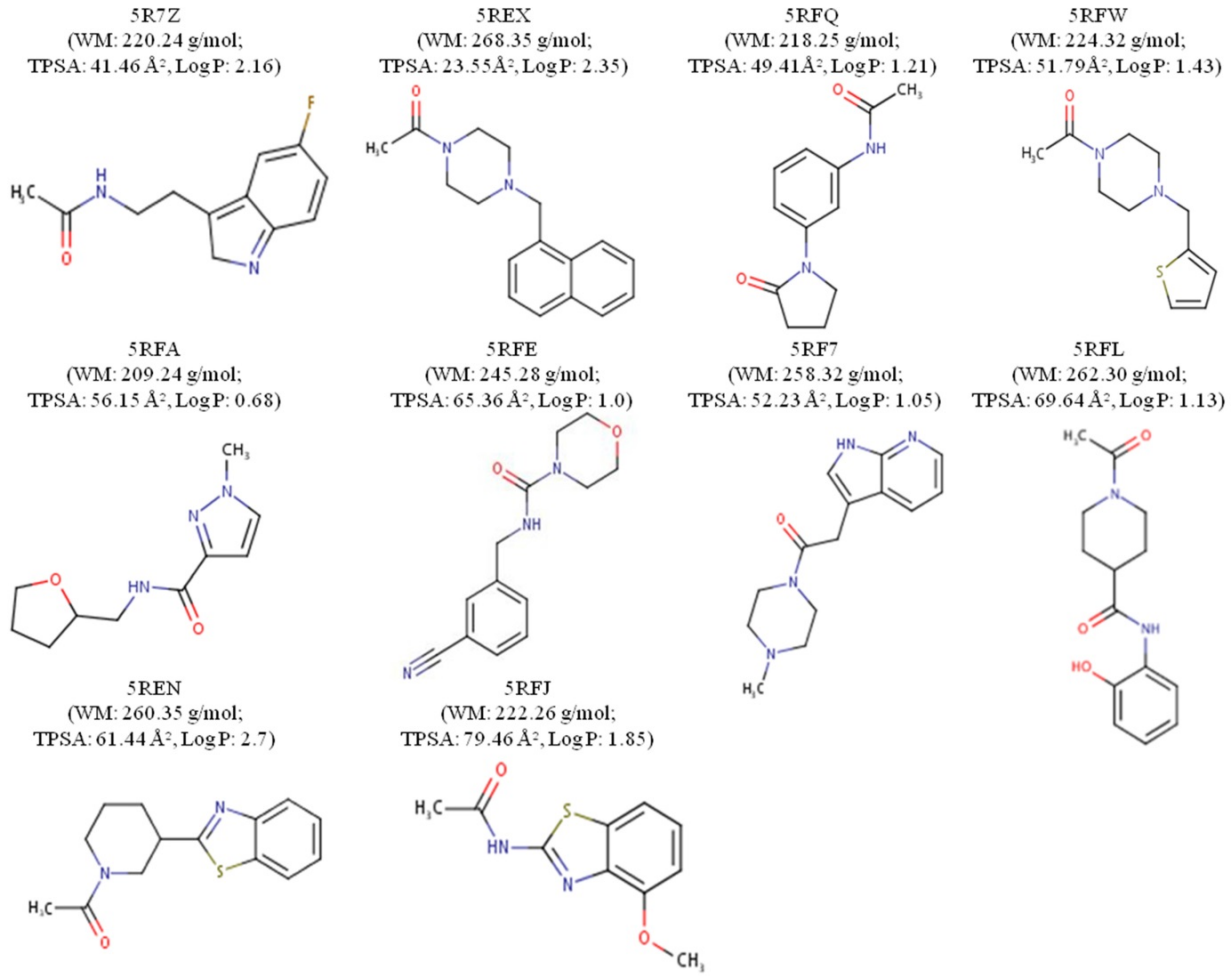
2D chemical structures and drug-likeness properties of the best 10 ligands measured through MVD.

The ligands were designated according to their PDB origin complex; for example, the 5RF7 complex ligand is to be mentioned as L5RF7. One can see in the structure of the molecules with better interaction energies (**Figure 2**) an amphoteric molecular pattern composed by the presence of aromatic rings (e.g. pyrrole, phenyl, indole and naphthalene) and by groups that can make electrostatic interactions (e.g. acetamide, oxopyrrolidine, hydroxyphenyl and pyrimidine). The aromaticity enables several interactions, such as *pi*-stacking (π-π stacking) with other aromatic rings or *pi*-alkyl and *pi*-anion, which is important in light of the presence of 4 histidines (His41, 163, 172 and 164), one phenylalanine140 and 2 methionines (Met49 and Met165) around the aromatic functional groups.

Properties such as the molecular hydrophobicity parameter LogP, molecular weight (MW) and the total polar surface area (TPSA) have been broadly used in modern rational drug discovery and design 46. The MW of the top 10 compounds (L5RF7, L5RFL, L5R7Z, L5REN, L5RFJ, L5REX, L5RFQ, L5RFW, L5RFA, L5RFE; **Figure 2**) varies in a short range from 209.24 to 268.35 g/mol. Conversely, the topological polar surface area (TPSA) values are from 23.55 Å^2^ to 79.46 Å^2^ and LogPs are between 0.68 and 2.70. Three compounds are lipophilic (L5R7Z, L5REX, L5REN); only compound L5RFA is rather hydrophilic with LogP 0.68 and PSAs of 56.16 Å^2^ 47. Among the many rules employed for pre-selection in the drug discovery phase, the most prominent one is Lipinski's rule-of-five (RO5) 48. It analyses molecular mass, LogP, number of hydrogen-bond donors and acceptors and the sum of nitrogen and oxygen atoms to predict absorption or permeation of a substance. None of the compounds violated RO5, which shows a promising characteristic as small molecule hits for these ligands and their analogs.

**Figure 3:**
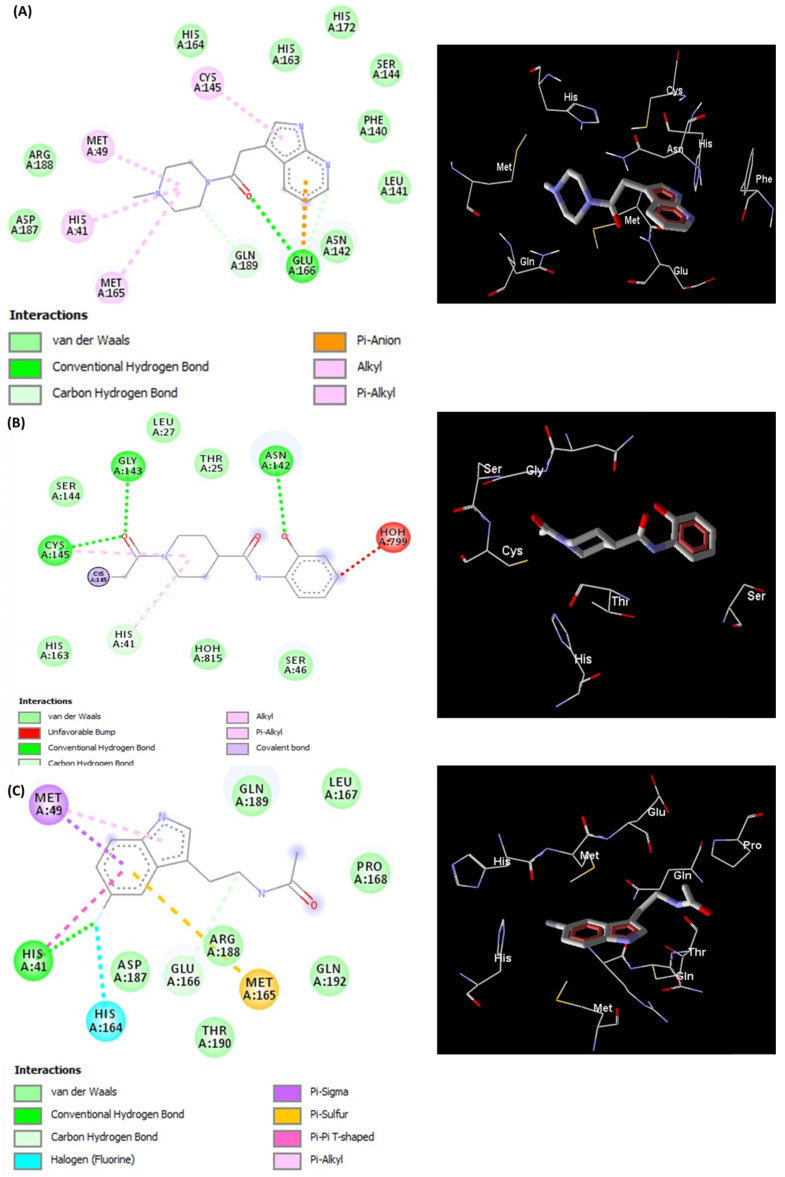
Representations of 2D and 3D interactions between Mpro (PDB id: 6Y84) residues and (**A**) L5RF7, (**B**) L5RFL, (**C**) L5R7Z ligands in the docked complex.

The docked pose of L5RF7 into the binding pocket of Mpro (**Figure 3A**) shows that the 4-methylpiperazine group binds in Met49, His41 and Met165, while a greater amount of interactions occur with aromatic moiety (pyrrolopyridine). Pi-anion and Pi-alkyl are formed between Glu166 and Cys145 with pyridine and pyrrol rings, respectively. Phe140, Leu141, Asn142, Ser144, His163 and His172 take part in a network of van der Waals interactions with pyrrolopyridine fragment. Hydrogen bonds are formed between Glu166 and the carbonyl oxygen atom from the linker.

The linker L5RFL (**Figure 3B**) showed an unfavourable bump with molecule HOH799 when docked with all structural water molecules. However, when removing the structural waters and redoing the docking study, there were no significant changes in the interaction energy. Hydrogen bonds exist between the Gly143, Cys145 and 1-acetyl-N groups, and between Asp142 and hydroxyphenyl. Despite the polar characteristic of the Thr25, Ser46, Ser144 and Hist163 residues, the ligand predominantly made van der Waals interactions due to the lack of groups capable to perform electrostatic interactions. Thus, the polar side chains of these residues were positioned across the ligand, *e.g.* Thr25, Ser46 and Ser144. The intramolecular interaction of 2-hydroxyphenyl and 4-carboxamide harms the potential to attract these polar residues to possible dipole-dipole or ion-dipole interactions, what must be analysed in future cases of structural planning.

L5R7Z (**Figure 3C**) showed an indole moiety that stacks with His41. This interaction is important because it brought together other residues of the binding site, i.e. Met49, Met165 and His164, which interacted positively with L5R7Z. One can see the interaction of the 5-fluorine group with histidines (His41 and His164) and that is due to the electrostatic potential generated by the inductive effects and by the resonance of the fluorine upon the indolic ring of the ligand and the imidazole ring of histidine. Leu 167, Pro168, Gln189 and Gln192 are involved in hydrophobic interactions with the linker (ethanamide group).

The Mpro amino-acid residues of CoV-2 show a high structural similarity with that of other viral ones present in different species of coronavirus, e.g. feline (PDB: 5EU8) and porcine (PDB: 4F49), or with previous COVIDs which overtook human beings (CoV-1, PDB: 4WME). The analyses made by our Blast and T-COFFEE servers have confirmed the high identity among these enzymes, with global score of 87 for the entire sequence analysed and 96 only for the restudies present in a 9Å radius of the Mpro binding cavities. Therefore, one can likely foresee that the COVID protease inhibitors are to have broad action spectra and with activity potential over future species of SARSr-CoV.

### Rational search for new protease inhibitor analogues with therapeutic potential against COVID-19

Molecular structure similarity searching used the cutoff criteria described in the method section and found 59 hit molecules from SwissSimilarity. These molecules have been prepared using Spartan and docking studies were performed on MVD and DS software on Mpro with unliganded active site (PDB: 6Y84).

**Figure 4:**
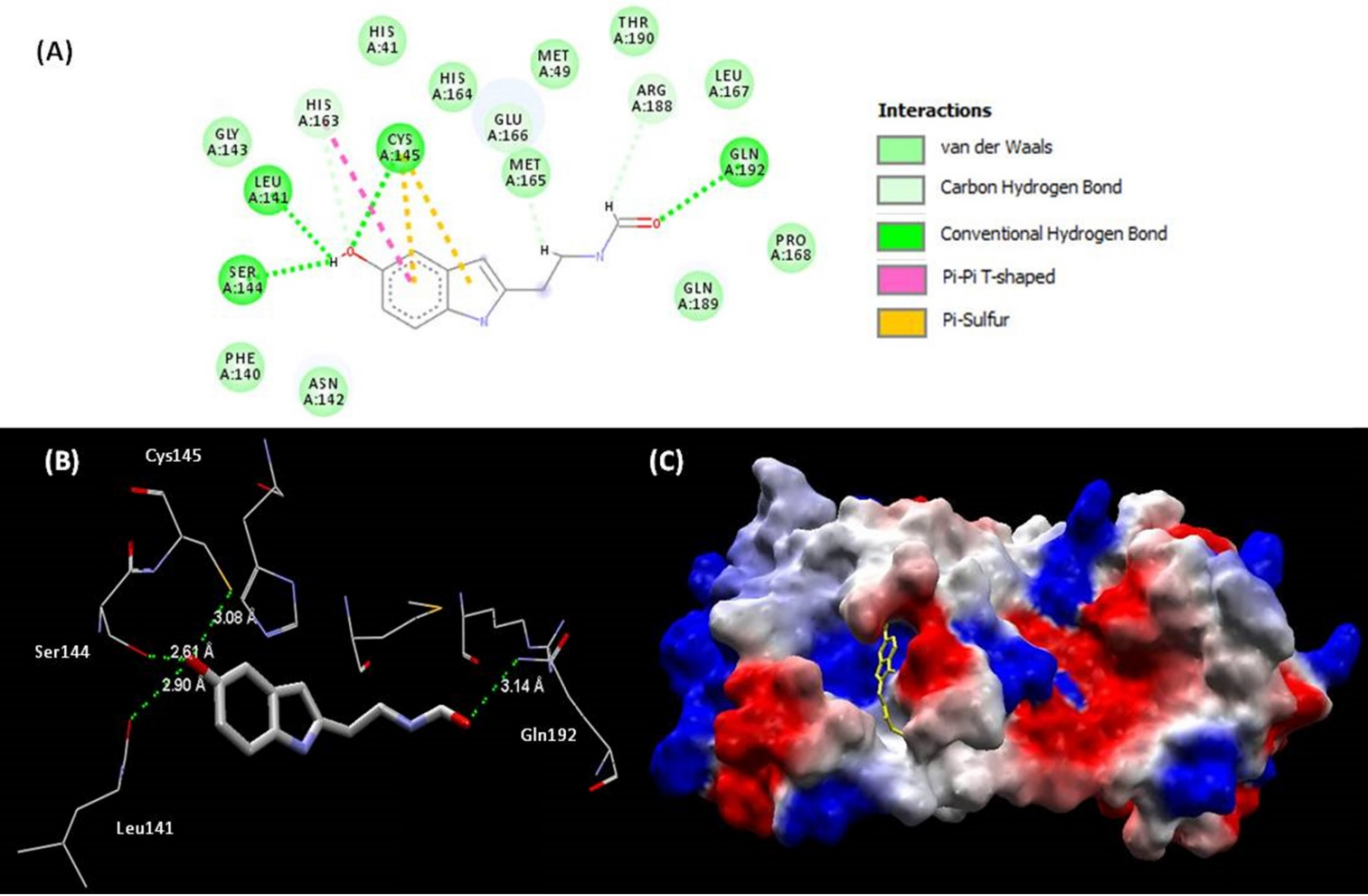
(**A**) Representations of 2D interactions between Mpro (PDB id: 6Y84) residues and melatonin in the docked complex. (**B**) Distance between melatonin and residues of Mpro and (**C**) mapping surface of electrostatic potential of this complex Mpro-melatonin (ligand highlighted in yellow).

All the 59 analogues bound inside the interaction site and 31 of them had better interaction energies than the 10 best evaluated complexes of PDB. Based on the docking energy results and previous studies of the bioactive characteristics of these analogues, one of them (melatonin) stood out and was selected for further analysis. The interaction between melatonin and Mpro (**Figure 4**) improved the values of binding energy and created a new perspective for a molecule with high therapeutic potential over the COVID-19 pathology to act, so far, only in more severe cases of the disease. The inhibitory effect over the SARS-CoV-2 main protease may be characterized as a big step towards the introduction of melatonin in the front line for the treatment.

#### Relationship between the infectious condition and the therapeutic potential of melatonin against SARS-CoV-2

**Figure 5:**
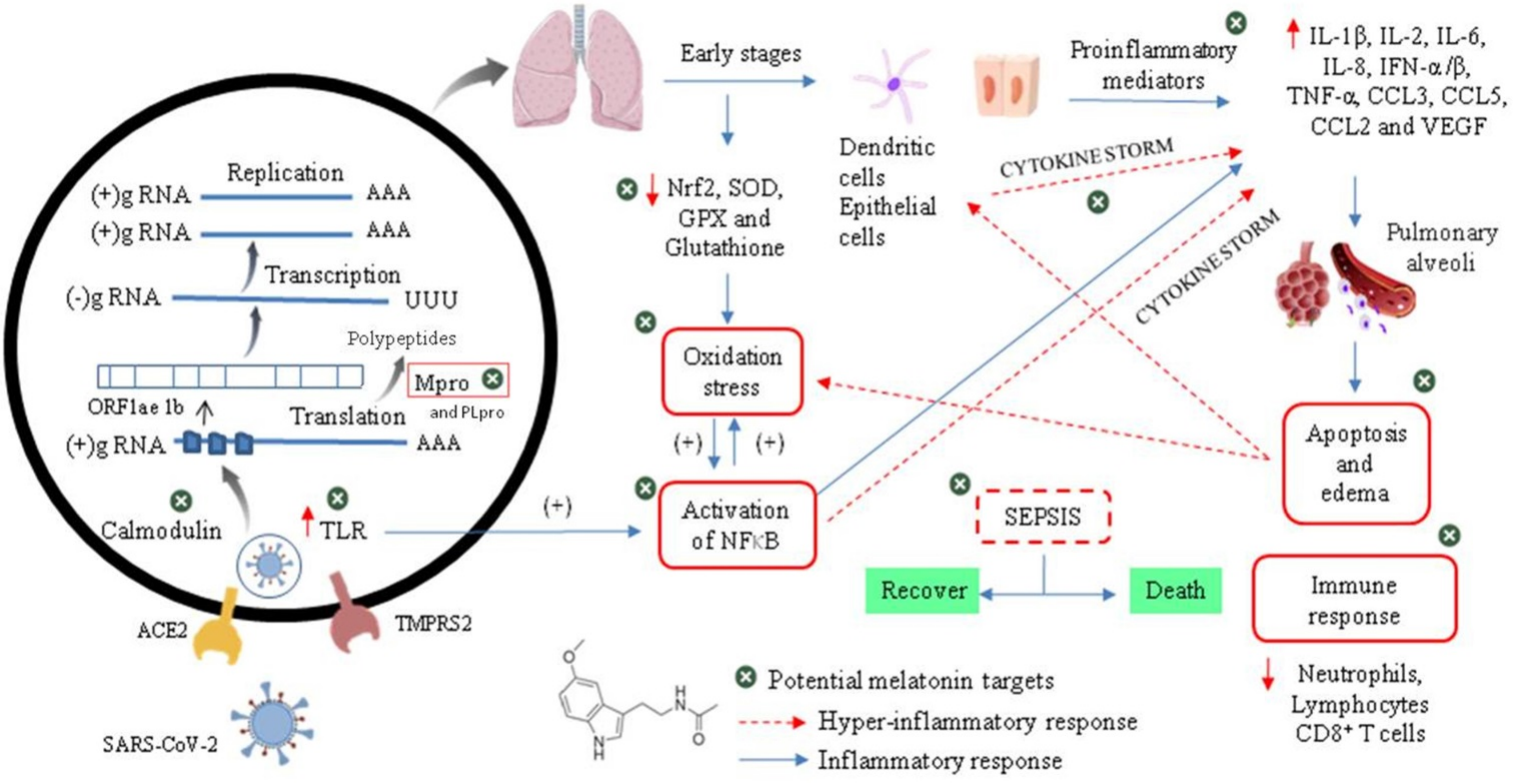
Viral cycle of CoV-2 and probable therapeutic melatonin targets.

To understand the need to clinically evaluate melatonin against Cov-2, we should make a brief introduction to infectious and physiopathological characteristics related mainly to the viral cycle and host immune response in the COVID-19 (**Figure 5**). As an emerging disease, these steps are not fully understood 49. It has been shown that the infection by SARS-CoV-2 is triggered by similar mechanisms of SARS-CoV and MERS-CoV, and it is amplified by dysfunctional immune responses 50.

The initial event of COVID-19 involves the infection of airway epithelial cells, alveolar epithelial cells, vascular endothelial cells, type-II pneumocytes and macrophages in the lung by SARS-CoV-2. Coronavirus glycosylated spike (S) protein binds to its cellular angiotensin-converting enzymes 2 (ACE2) receptor leading to the membrane fusion between the virus and human plasma membrane 51. Besides that, this invasion process is facilitated by a transmembrane serine protease 2 (TMPRSS2) produced by the host cell. These interactions are considered as critical for cell entry and future viral replication 52, 53. After membrane fusion, the viral RNA genome is released into the cytoplasm and is translated in two polyproteins (pp1a and pp1ab from ORF1a and 1b), which are cleaved by Mpro and papain-like protease (PLpro). This cleavage results in 15 new non-structural proteins (nsp) that compose the replication-transcription complex. Then, the viral genome begins to replicate and generate individual sub-genomic mRNA templates needed for the translation of the viral structural and accessory proteins. The newly formed RNA, nucleocapsid proteins and envelope glycoproteins assemble to form viral particles mediated by the endoplasmic reticulum and Golgi apparatus. Finally, vesicles containing the virus particles are exocytosed from the host cell to continue the infection cycle, resulting in the death of surrounding cells and tissue injury 54.

Effective host immune response against CoV-2 infection begins when viral RNAs, as pathogen-associated molecular patterns (PAMPs), are recognized by the pattern recognition receptors (PRRs) in residing antigen-presenting cells, such as macrophages and dendritic cells. Thus, PAMPs are sensed by PRRs, such as the RIG-I-like receptors (RLRs) and Toll-like receptors (TLRs), initiating a downstream signalling cascade and activating the transcription factors IRF3, IRF7 and NF-kB. This leads to a broad induction of pro-inflammatory genes, particularly those encoding Type I interferon (IFNs), cytokines and chemokines. Moreover, NF-kB also induces the expression of a large number of genes involved in inflammation, stress, proliferation and apoptotic responses, such as cyclooxygenase 2 (COX-2), nitric oxide synthase 2 (NOS-2), vascular endothelial growth factor (VEGF), adhesion molecules, immune-receptors and growth factors. This inflammatory state stimulates local upregulation of adhesion molecules and the creation of chemotactic gradients, and also increases vascular permeability (mediated by VEGF), which together promotes the extravascular recruitment and activation of defence cells 55. Subsequently, lymphocytes (such as NK, CD4+ T, CD8+ T and B cells) are recruited into the lung to eradicate the pathogen by killing virus-infected cells and producing virus-specific antibodies 56. The overproduction of these chemokines and cytokines may contribute to the control of infection or to the development of critical prognostics.

Although most of the cases (about 80%) are asymptomatic or mild, clinical manifestations include fever, dry cough, shortness of breath, muscle ache, dizziness, headache, sore throat, rhinorrhoea, chest pain, diarrhoea, nausea and vomiting. Nevertheless, an uncontrolled immune response can trigger a cytokine storm and a large amount of free radicals, resulting in severe damages to the lungs, kidneys, heart, followed by possible sepsis, multiple organ failure and death 57. Patients with severe COVID-19 cases exhibit elevated serum levels of proinflammatory cytokines, i.e., interleukins- (IL-6, -1β, -2, -8, -17, -10 and -4), interferon γ (IFN-α/β), granulocyte colony-stimulating factor (G-CSF), granulocyte-macrophage colony-stimulating factor (GM-CSF), interferon-inducible protein (IP-10), monocyte chemoattractant protein 1 (MCP1), macrophage inflammatory proteins 1α (MIP1α) and tumour necrosis factor α (TNF-α). In this intensity of disease, lymphopenia is a common feature, with drastically reduced numbers of CD4+ T cells, CD8+ T cells, B cells and natural killer (NK) cells, as well as a reduced percentage of monocytes, eosinophils and basophils. An increase in neutrophil-to-lymphocyte ratio may indicate poor clinical outcome.

There is also a massive infiltration of neutrophils and macrophages in the lung and excess of fluid into the alveoli, resulting in severe acute respiratory syndrome (SARS). During this advanced stage of infection, there may be a reduction in the levels of surfactant protein transcription, which generates increased pulmonary surface tension, reduced respiratory capacity and increased risk of respiratory collapse during expiration 58. In addition, pathogenic examination of the lung showed proteinaceous exudate with globules, inflammatory cellular infiltration, bilateral diffuse alveolar damage with edema, pneumocyte desquamation and hyaline membrane formation.

There is a correlation between serum levels of cytokines and the severity of the disease indicating that mortality might be due to virally-driven hyper-inflammation. Interestingly, some COVID-19 patients, in spite of being negative for the viral nucleic acid test, have a high level of inflammation, which indicates that this factor is even more important than the viral load to define the patient's prognosis. Hyper-inflammation promotes cellular apoptosis and necrosis in various tissues, which would further trigger inflammation, followed by the release of more inflammatory factors, increased vascular permeability, mobility and accumulation of monocytes, macrophages and neutrophils. This indicates that if an exacerbated inflammatory process is not controlled, a vicious circle is created, capable of activating the "cytokine storm" and a possible evolution to sepsis 59.

In addition to the overwhelming immune response, SARS-CoV-2 can target organs that express ACE2, including the heart, kidney and gastrointestinal tract, aggravating the case. High levels of NOS-2-derived nitric oxide (NO), superoxide and other products of inflammation drive the body to a hemodynamic collapse, including impaired vascular permeability, vasodilatation, tissue hypoperfusion and untreatable hypotension. Thus, tissue hypoxia and the elevated production of reactive oxygen species (ROS) compromise mitochondrial function and activate cell death pathways, increasing tissue damage and the formation of fibrosis in target tissues, which express ACE2, including the heart, kidney and gastrointestinal tract 60-62. Therefore, the unsolved infection becomes a life-threatening condition evolving to sepsis and even death.

### Melatonin

Melatonin (N-acetyl-5-methoxytryptamine) is an endogenous molecule, the final metabolite of the melatoninergic pathway from serotonin to N-acetylserotonin (NAS) by arylalkylamine N-acetylserotonin (AA-NAT), with NAS then converted to melatonin by hydroxyindole O-methyltransferase (HIOMT). For a long time, science has only discussed the properties of melatonin in the control of the circadian cycle in the absence of light and its biosynthesis by the pineal gland. Therefore, it is called as the hormone of darkness 63. Nevertheless, it is noteworthy that melatonin is also produced by immune cells after NF-ΚB activation, a transcription factor involved in controlling the expression of several genes linked to the inflammatory response. This observation included melatonin as an integral part of the host defense system, where it has the autocrine and paracrine action that dampens immune responsiveness, while increasing the anti-inflammatory and phagocytic response in immune cells 64, 65.

It has been proven that there is a positive correlation between aging and the reduction of plasmatic melatonin, what may affect sleep quality as well as the suitable control of inflammatory processes and thus a higher risk of hyper-inflammation 66. The beneficial effect of melatonin on acute lung injury states, sepsis and hyper-inflammatory processes caused by viruses, bacteria or radiation has been previously documented 67-70. Accordingly, it is possible to imagine that melatonin can be indicated as adjuvant therapy for the treatment of COVID-19 due to its potential cytoprotective, anti-inflammatory, immunosuppressive and, probably, antiviral mechanisms 64. Another relevant topic is that the drug interaction of melatonin with the main drugs currently available can reduce the side effects such as renal damage and oxidative stress of the lopinavir/ritonavir association, potentiate the antiviral response of ribavirin and reduce oedema when associated with methylprednisolone 66.

Anti-inflammatory effect of melatonin occurs through a range of mechanisms and mediators. During inflammatory and immunological processes, the intracellular mediator NF-ΚB (activated by the degradation of its inhibitor IKB by ROS pro-inflammatory cytokines) plays a key role in regulating multiple aspects of the innate and adaptive immune response, and acts as a signal for the expression of pro-inflammatory reaction, inflammasomes and immune cells (e.g. macrophages). These cells are elevated during hyper-inflammation and are pivotal in the production of pro-inflammatory cytokines and chemokines involved with a higher inflammatory response and worse prognosis. Melatonin could modulate inflammation by decreasing the activation of both NF-κB and inflammasomes NLRP3 (nucleotide-binding domain leucine-rich repeat (NLR) and pyrin domain-containing receptor 3). Therefore, there will be a reduction in cell membrane damage and the inflammatory release of cell content to the extracellular space 66. The mechanism of action as inhibitor of NF-κB has not yet been fully clarified but it seems to involve reducing oxidative stress via NF-E2-related factor (Nrf2), reducing the binding ability of NF-κB via acetylation of the p50 subunit 71-73 and toll-like receptors (TLRs). It inhibits, under inflammatory conditions, the expression of TLR2, TLR4 and TLR9, key mediators of the innate immune response that are massively activated when the virus reacts with its endosomal receptors or in pro-inflammatory processes 65.

Melatonin can up-regulate Nrf2, an important transcription factor activated by oxidative stress. It has a role in the regulation of the expression of detoxifying and antioxidant genes, inducing phase II protective gene, such as heme oxygenase-1 (HO-1), glutathione-S-transferase (GST) and UDP-glucuronosyltransferase (UGT) 1A1, which mediate cell survival and mitochondrial dose-dependent damage. Concomitantly, a more intense induction of inflammatory biomarkers was observed in Nrf2 KO mice, i.e. IL-1β, IL-6, TNFα and pro-inflammatory enzymes iNOS and COX2. Therefore, the increase in Nrf2 by melatonin is crucial in tissue protection, such as pulmonary, hepatocytes, renal and cardiac in patients positive for SARS-CoV-2 74, 75.

Asadi-Pooya & Simani 76 highlighted that coronavirus infections have been associated with neurological manifestations (e.g. febrile seizures, convulsions, change in mental status and encephalitis), causing inflammation and demyelination. The effect of melatonin in experimental autoimmune encephalomyelitis has demonstrated a therapeutic role by ameliorating the clinical severity and restricting the infiltration of inflammatory Th17 cells into the CNS. Moreover, another study reinforces this potential therapeutic by confirming an enhancement of IL-10, a multifunctional cytokine with expression of antiviral and cytoprotective properties 77 and, in contrast, that also suppresses IFN-γ, IL-17, IL-6, CCL20 and T cell proliferation in the CNS 78.

Severe COVID-19 patients may develop sepsis that is characterized by uncontrolled increase in oxidative stress, mitochondrial dysfunction, cellular energy failure, vasodilatation with hypo-responsiveness to drugs and multi-organ failure. Melatonin acts as a free radical scavenger and stimulates anti-oxidative enzymes (e.g., superoxide dismutase) and HO-1 (via activation of Nrf2), and also impairs pro-oxidative enzymes (e.g. iNOS) with a consequent reduction of peroxynitrite formation 79. Several studies have demonstrated the therapeutic efficacy of melatonin in controlling septic conditions induced by toxic drugs or infection 80-82.

Another important anti-inflammatory effect is the inhibition of bradikinin-induced vasodilation, as well as the reduction of vascular permeability. It occurs since the melatonin treatment suppresses the vascular endothelial growth factor (VEGF) mRNA and decreases hypoxia-inducible factor (HIF)-1alpha protein levels and expression 82. These changes added to the reduction of adhesion molecules (by inhibition of NF-κB) allow one to predict a lesser leukocyte adhesion to endothelial cells and to decrease migration into tissues, especially alveoli 65.

Moreover, melatonin has possible anti-infectious activity through different mechanisms. It can promote the formation of the neutrophil extracellular trap (NET), increase levels of the generally beneficial cells Th1 and NK cells. Previous studies with virus-infected animal models support the indication of melatonin as an antiviral after observing a reduction in viral load, improving the functions of the infected organ, decreased mortality and viremia. However, despite the reported viral load reductions, these studies have not discussed the possible mechanism involved in these outcomes. Supported by our results presented in the possible inhibition of Mpro and interaction with calmodulin (previous studies), we propose two new possible mechanisms in addition to the properties already reported to control hyper-inflammation and thus control COVID-19 67.

ACE2 is a type I transmembrane metallopeptidase with an extracellular ectodomain containing its zinc-coordinating catalytic site that is expressed and active in most tissues, mainly lung, heart, brain and kidney. Cell membranes richer of ACE2 become more susceptible to CoV-2 infection. Regulation of its expression at the cell surface is therefore of prime importance to control of virus-induced cell fusion by host 83, 84.

Calmodulin (CaM) is considered as the major regulator of Ca^2+^-dependent signalling in all eukaryotic cells. CaM regulated the surface expression and retention of ACE2 in the plasma membrane. Inhibitors of this calcium binding protein enhance the release of the ACE2 ectodomain and decrease the association between CaM and ACE2 in a dose- and time-dependent manner 85. *In vitro* studies have demonstrated that melatonin binds to CaM (Kd of 188 pM) and inhibits it in a Ca^2+^-dependent pocket 86, 89. As a consequence of this interaction, melatonin can be classified as an indirect inhibitor of ACE2-CoV2 coupling during viral particle fusion 89. Both, melatonin and CaM, are phylogenetically preserved. Therefore, their interaction probably stands out as an important pathway for the regulation of cell physiology 90, including protection against CoV-2 infection, autophagy, cell proliferation and apoptosis via inflammatory process, etc. 91.

Viral proteases are responsible for vital processing polyproteins, leading to the formation of structural and functional viral proteins. Since Mpro is unique in the virus and not found in the host cells, this protein is a prominent target for the development of antivirals against coronavirus infections. Mpro is a key CoV enzyme, which plays a crucial role by cleaving viral polyproteins during coronavirus replication and transcription 92. The inhibition of viral protease is a strategy used successfully in many viral infections, mainly against the human immunodeficiency virus (HIV). It results in the incapacity of the new viral particles formed to replicate, producing, after the budding stage, only non-infectious virions.

Mpro is composed by 3 domains (I-chymotrypsin, II-picornavirus 3C protease-like and III-globular cluster involved in protein dimerisation). We can observe in this study that melatonin interacts in the site between domains I and II, blocking the access to the catalytic cysteine 145 (Fig. 4B) 92. The binding site of melatonin on SARS-CoV-2 Mpro is considered as highly conserved, 96% identical (98% similar) in the aminoacid sequence to that of SARS-CoV Mpro. After comparing the binding site of melatonin to Mpro active site of SARS-CoV-2 and other coronavirus, we can confirm that melatonin is a potential inhibitor of this protease (Fig, 4A). The residues His41, Phe140, Ser144, Cys145, His163, His164, Glu 166, Gln189 and Thr190 are important fo the interaction of inhibitors of SARS-CoV Mpro 93 and are also present on the binding site of melatonin. Inhibitory actions on calmodulin and Mpro can further expand the spectrum of action of melatonin 92 and help to explain some antiviral results.

Viruses cause host mitochondrial dysfunction, which can reduce melatonin biosynthesis. The reduced serum melatonin cannot upregulate the expression of antioxidant enzymes, control the immune response, oxidative stress and the outcome of hyperinflammation. Therefore, the administration of melatonin has been successfully used to treat different viral infections in animal models, *e.g.* parvovirus, Venezuelan equine encephalomyelitis virus, LP-BM5 retrovirus, respiratory syncytial virus and rabbit hemorrhagic disease virus 65, 94.

A recent study determined the efficacy and tolerability of high-dose melatonin (36 mg/day to 72 mg/day per os (p.o.) in 4 divided doses) as adjuvant therapy, in addition to standard and/or empirical therapy 95. All the patients were admitted with flu-like symptoms and chest imaging findings of ground glass opacities highly suggestive of COVID19 pneumonia. The 10 patients given melatonin had high-risk features determined for age (> 60 years) or/and established comorbidities. No significant side-effects were noted except for drowsiness. Benefits of time were observed for clinical improvement (reduction of symptoms, stabilization and/or regression of lung infiltrates, decrease in pro-inflammatory markers), as well as the need for mechanical ventilation, duration of hospital stay and outcome (death, or recovery and discharge) 95. These results may encourage new greater trial studies.

In addition to its therapeutic properties on hyper-inflammation, oedema, immune dysregulation and possible sepsis in moderate-advanced stages of the disease, this drug may also be evaluated for its antiviral action in more recent stages of COVID-19 infection. Therapeutic regimes for prophylaxis and COVID-19 treatment using melatonin either as monotherapy or associated were proposed, i.e., 50- and 300-mg daily doses twice a day for 7 days for the control of both light and severe cases of COVID-19, respectively 96,97. They have considered only the therapeutic potential over the modulation of the immune and inflammatory responses, not mentioning the new mechanisms proposed in the present study on the inhibition of calmodulin and Mprotease. Those doses still have to be validated to measure the efficacy and clinical outcomes.

## Conclusion

We are dealing with one of the most potentially deadly pandemics in terms of economic and social damages of the last century. In the absence of readily accessible and efficient medicines, there is an urgent need for the development of some treatment. Therefore, finding a substance with therapeutic potential, low toxicity and cost, with mechanisms of action described, is certainly a great step in the therapeutic advance thanks to its wide use.

The results presented provide the first indication that the therapeutic administration of melatonin can be an effective Mpro inhibitor to prevent viral replication by binding to viral protease. Thus, we suggest that melatonin could play an adjunct therapeutic role in treating COVID-19 in different stages of the disease. Therefore, clinical studies on the possible therapeutic value of melatonin on this infection should be performed in the near future.

## Figures and Tables

**Table 1:** Docking calculations depicting the PDB code, binding site volume (Å) and binding energy (calculated by the Moldock Score Grid algorithm, Kcal/mol) obtained from MVD for 74 ligand-Mpro complexes. The 10 complexes that showed the best interaction energy (more negative) are highlighted in bold. The complexes highlighted in the gap with the asterisk (*) bore their ligands out of the interaction site of the protease, with interaction below -60 Kcal/mol.
